# Metabolite Signature of Physical Activity and the Risk of Type 2 Diabetes in 7271 Men

**DOI:** 10.3390/metabo12010069

**Published:** 2022-01-12

**Authors:** Susanna Maria Kemppainen, Lilian Fernandes Silva, Maria Anneli Lankinen, Ursula Schwab, Markku Laakso

**Affiliations:** 1 Institute of Public Health and Clinical Nutrition, University of Eastern Finland, 70210 Kuopio, Finland; susanna.m.kemppainen@uef.fi (S.M.K.); maria.lankinen@uef.fi (M.A.L.); ursula.schwab@uef.fi (U.S.); 2 Institute of Clinical Medicine, Internal Medicine, University of Eastern Finland, 70210 Kuopio, Finland; lilian.fernandes.silva@uef.fi; 3 Department of Medicine, Endocrinology and Clinical Nutrition, Kuopio University Hospital, 70210 Kuopio, Finland; 4 Department of Medicine, Kuopio University Hospital, 70210 Kuopio, Finland

**Keywords:** physical activity, type 2 diabetes, insulin sensitivity, insulin secretion, metabolites, healthy diet

## Abstract

Large population-based studies investigating the association of physical activity (PA) with the metabolite signature contribute significantly to the understanding of the effects of PA on metabolic pathways associated with the risk of type 2 diabetes. Our study included 8749 Finnish men without diabetes at baseline recruited from the Metabolic Syndrome in Men (METSIM) cohort. We used a questionnaire to measure leisure-time PA. Metabolites were measured in 7271 men as a part of Metabolon’s untargeted Discovery HD4 platform using ultrahigh-performance liquid chromatography–tandem mass spectrometry. We found 198 metabolites significantly associated with PA. Several of these metabolites were novel including especially steroids, amino acids, imidazoles, carboxylic acids, and hydroxy acids. Increased PA was significantly associated with high levels of choline plasmalogens, lysophosphatidylcholines, polyunsaturated fatty acids, carotenoids, long chain acylcarnitines, imidazoles, bilirubins, aryl sulfates, hydroxy acids, indolepropionate, and indolelactate. Several of these metabolites have been previously associated with a decreased risk of type 2 diabetes and with a healthy diet. Our population-based study shows that the metabolite signature of increased PA includes multiple metabolic pathways and is associated with better adherence to a healthy lifestyle.

## 1. Introduction

Type 2 diabetes has reached epidemic dimensions worldwide [1]. Obesity and lack of physical activity (PA) are important risk factors for the development of type 2 diabetes [2]. Lifestyle intervention studies have shown that PA and a healthy diet prevent the conversion to type 2 diabetes in individuals with high risk of this disease [3]. Similarly, prospective cohort studies have reported that PA reduces the risk of type 2 diabetes by 30–50% compared to those who are physically inactive [4]. The current recommendation to prevent type 2 diabetes is PA at least 150 min/week at moderate intensity [5]. 

PA has been shown to reduce glucose levels and increase insulin sensitivity [6]. A single bout of exercise enhances skeletal muscle glucose uptake not only via an insulin-dependent pathway but also via mechanisms that are independent of insulin [6]. To maintain the beneficial effects of PA, it should be regular, since the effect of a single bout lasts only about 48 h [5,6]. The effect of PA on insulin secretion is poorly understood [7]. In many studies, there are also limitations in the methods to measure insulin sensitivity and insulin secretion [8]. Conversion to type 2 diabetes occurs when insulin secretion cannot compensate for insulin resistance in peripheral tissues.

High-throughput metabolomics studies have revolutionized biomarker research. The present key technologies for metabolomics are mass spectrometry and proton nuclear magnetic resonance spectroscopy (NMR). Mass spectrometry plays an increasingly dominant role in the metabolomics field. It is a highly sensitive method for detection and quantitation, and can identify hundreds to thousands of metabolites, whereas proton NMR has low sensitivity and can identify only about 100 metabolites [9]. 

Previous observational studies on the association of PA with metabolites have often been small and heterogeneous [10]. Ding et al. published the largest study so far on the relationship between PA and metabolites including 5197 participants from the Nurses’ Health Study, Nurses’ Health Study II, and the Health Professionals Follow-up Study. They identified 20 metabolites associated with PA, including two amino acids, four cholesteryl esters, and eight phosphatidylcholines and lyso-phosphatidylethanolamines [11]. However, this study did not investigate the association of PA with metabolites in relation to the risk of type 2 diabetes.

In the present study, we investigated the association of insulin sensitivity, insulin secretion, and metabolite signature of PA with the risk for type 2 diabetes in a large prospective population-based cohort in Finland. 

## 2. Materials and Methods

### 2.1. Subjects

The METabolic Syndrome In Men (METSIM) cohort includes 10,197 Finnish men randomly selected from the population register of Kuopio, Eastern Finland, aged 45–73 years (58 ± 7 years, mean ± SD) [12]. The current study includes 8749 participants without diabetes at baseline. Glucose tolerance was evaluated by a 2-h oral glucose tolerance test according to the ADA criteria [13]. At baseline, 3034 (29.8%) of all participants had normal glucose tolerance, 4344 (42.6%) had isolated impaired fasting glucose, 312 (3.1%) had isolated impaired glucose tolerance, 1059 (10.3%) had both impaired fasting glucose and impaired glucose tolerance, and 1437 (14.1%) had diabetes. We excluded from statistical analyses participants with type 1 diabetes (*n* = 25) and type 2 diabetes at baseline (*n* = 1412) and participants having missing data from an oral glucose tolerance test (*n* = 11). We identified 1153 incident cases of type diabetes after a mean of 7.8-year follow-up. Type 2 diabetes diagnosis was based on fasting plasma glucose ≥7.0 mmol/L, 2-h plasma glucose ≥11.1 mmol/L, glycated hemoglobin (HbA1c) ≥6.5% (≥48 mmol/mol) or antidiabetic medication started between the baseline and the follow-up visits. 

### 2.2. Physical Activity Questionnaire and Other Lifestyle Factors

We evaluated leisure-time PA at baseline and follow-up visits by a questionnaire used in the Mini-Finland Health Survey [14]. The questionnaire measures frequency and duration of PA on a 4-point scale. The category PA1 means little or no PA, PA2 physical activity in context of other hobbies or physical activity occasionally, PA3 at least 30 min of physical activity regularly ≤2 times a week, and PA4 at least 30 min of physical activity regularly ≥3 times a week. The physically inactive group (PA-) includes the PA1 and PA2 categories and the physically active group (PA+) includes the PA3 and PA4 categories. We generated three categories for PA changes during the follow-up compared to the baseline study: an increase of PA (PA^Inc^), a decrease of PA (PA^Dec^), and no change in PA (PA^0^).

### 2.3. Clinical and Laboratory Measurements

Measurements of height and weight have been described previously [12]. An oral glucose tolerance test was performed (75 g glucose) and blood samples were collected at 0, 30, and 120 min to measure plasma glucose and insulin concentrations. We recorded information about alcohol consumption (g/week) and smoking. Plasma glucose concentration was determined using an enzymatic hexokinase photometric assay (Konelab Systems reagents, Thermo Fisher Scientific; Vantaa, Finland). Insulin was assessed by immunoassay (ADVIA Centaur Insulin IRI no. 02230141; Siemens Medical Solutions Diagnostics, Tarrytown, NY, USA). 

### 2.4. Measurement of Metabolites

Non-targeted metabolomics profiling was performed at Metabolon, Inc. (Durham, NC, USA) on EDTA-plasma samples obtained after an overnight fast. Briefly, methanol extraction of biochemicals followed by a non-targeted relative quantitative liquid chromatography–tandem mass spectrometry (LC-MS/MS) Metabolon DiscoveryHD4 platform was applied to assay 1260 named metabolites from 7271 participants. Samples were randomized across batches. Batches contained ~144 METSIM samples and 20 well-characterized human-EDTA plasma samples for quality control. All samples were processed together for peak quantification and data scaling. We quantified raw mass spectrometry peaks for each metabolite using the area under the curve and evaluated overall process variability by the median relative standard deviation for endogenous metabolites present in all 20 technical replicates in each batch. We adjusted for variation caused by day-to-day instrument tuning differences and columns used for biochemical extraction by scaling the raw peak quantification to the median for each metabolite by the Metabolon batch. 

### 2.5. Calculations

The Matsuda insulin sensitivity index (Matsuda ISI) was calculated as previously reported [15,16], and the insulin secretion index (InsAUC0–30 /GluAUC0–30) was calculated as follows (insulin at 0 min + insulin at 30 min)/(glucose at 0 min + glucose at 30 min). We have previously validated Matsuda ISI as the most reliable index for insulin sensitivity compared with the M value of the euglycemic hyperinsulinemic clamp, and InsAUC0–30/GluAUC0–30 as the most reliable marker of insulin secretion, as compared with insulin secretion during a frequently sampled intravenous glucose tolerance test [12]. We calculated the disposition index (DI), a measure of insulin secretion adjusted for prevailing insulin sensitivity, as Matsuda ISI × (InsAUC0–30/GluAUC0–30) [12].

### 2.6. Statistical Analyses

We performed statistical analyses using IBM SPSS, version 27 (IBM Corp., Armonk, NY, USA). We logarithmically transformed all variables due to their skewed distributions, except for age and follow-up time. We used one-way ANOVA to assess the differences in clinical traits and metabolites between the (PA−) and the (PA+) groups. We examined the associations of PA with glucose and insulin concentrations, insulin sensitivity, and insulin secretion at baseline with linear regression adjusted for age, smoking (current smoker vs. non-smoker), alcohol consumption, and body mass index (BMI). In prospective linear regression analyses we adjusted for age, corresponding metabolic trait at baseline, follow-up time (in months), BMI, smoking, and alcohol consumption. We applied Cox regression to investigate the association of PA with incident type 2 diabetes after adjustment for age, BMI, smoking, and alcohol consumption.

## 3. Results

### 3.1. Baseline Clinical and Laboratory Characteristics of the Participants in the Categories of Physical Activity

BMI, smoking, and alcohol consumption were significantly lower in participants in the physically active groups (PA4, PA3) than in the physically inactive groups (PA2, PA1) (Table 1). 

At baseline, fasting and 2 h glucose and insulin concentrations were significantly decreased, and insulin sensitivity (Matsuda ISI) and insulin secretion (Disposition index) was significantly increased in the highest (PA3, PA4) PA categories (Table 2). After the adjustments for confounding variables, the difference in fasting glucose across the PA categories lost its statistical significance. 

### 3.2. Physical Activity and Incident Type 2 Diabetes 

In unadjusted statistical analysis increased PA was associated with a decreased risk of type 2 diabetes (PA3, HR 0.70, 95% confidence intervals, CI, 0.55, 0.89, *p* = 0.004; PA4, HR 0.61, 95% CI, 0.49, 0.75), *p* < 0.001). After the adjustment for age, BMI, smoking, and alcohol consumption, the association with the risk of type 2 diabetes was non-significant (Table 3). 

### 3.3. PA Changes during the Follow-Up

A total of 50% of the follow-up participants at baseline and 60% at follow-up reported engaging in PA at least 90 min per week (category PA4). Of the participants at follow up, 27% increased their PA (PA^Inc^, *n* = 1597), 55% did not change (PA^0^, *n* = 3230), and 18% decreased their PA (PA^Dec^, *n* = 1040). The majority (78%) of those who belonged to the highest activity category (PA4) at baseline did not change their PA. Of those who had no or little PA (category PA1) at baseline (5%), 29% did not change PA, 43% increased PA by one level (category PA2), 7% increased PA by two levels (category PA3) and 21% increased PA by three levels (PA4) during the follow-up. 

### 3.4. Association of PA Changes with Glucose and Insulin Concentrations, Insulin Sensitivity, and Insulin Secretion at the Follow-Up 

**Figure 1:**
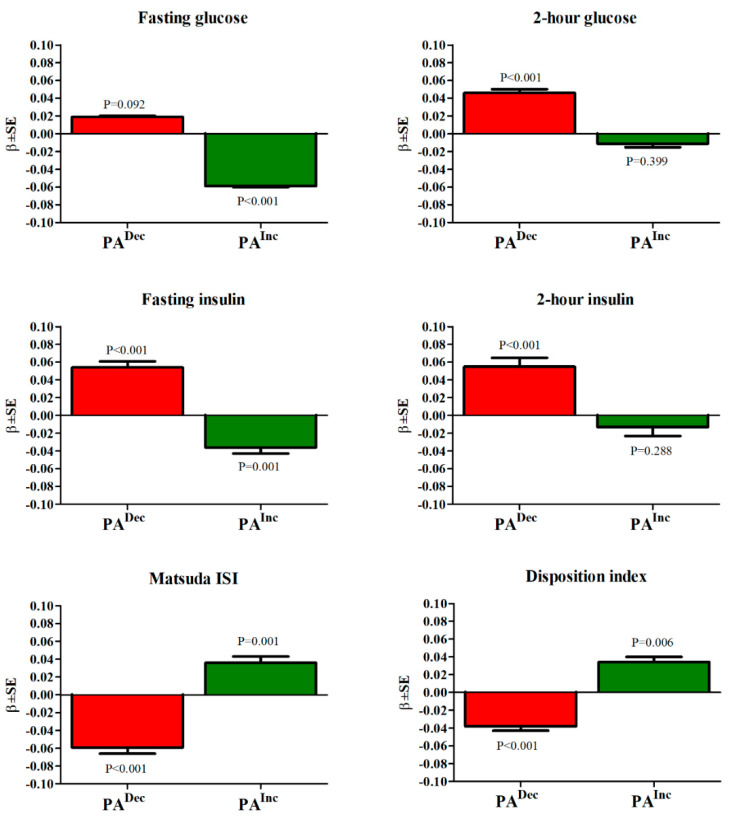
The association of physical activity (PA) changes with glucose and insulin concentrations, insulin sensitivity, and insulin secretion in 5867 participants without diabetes at baseline, subjected to oral glucose tolerance tests both at baseline and follow-up visits. The effect sizes (β, SE) are given as the standardized mean differences for participants who decreased their PA (PA^Dec^) or increased their PA (PA^Inc^) compared to the reference category of no changes in their PA (PA^0^). The *p-*values were adjusted for age, follow-up time, corresponding metabolic trait at baseline, BMI, smoking, alcohol consumption, and PA at baseline.

Participants who decreased their PA had a significant increase in their fasting and 2 h glucose, fasting insulin, and 2 h insulin concentrations and a decrease in their insulin sensitivity and insulin secretion compared to those who did not change their PA (Figure 1). Participants who increased their PA had a significant decrease in fasting glucose and insulin concentrations and an increase in insulin sensitivity and insulin secretion. 

### 3.5. Metabolites Associated with Physical Activity

**Figure 2:**
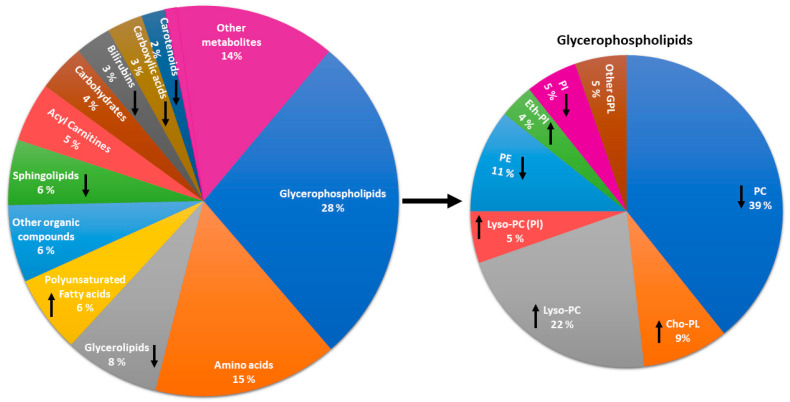
Metabolite groups having statistically significant difference between participants with high physical activity and participants with low physical activity. Abbreviations: GPL, glycerophospholipids; Lyso-PC, lysophosphatidylcholine; Lyso-PL-Cho, lysoplasmalogen-choline; PC, phosphatidylcholine; PE, phosphatidylethanolamine; Pl-Cho, plamalogen-choline; Pl-Eth, plasmalogen-ethanolamine; PI, phosphatidylinositol.

Figure 2 shows the metabolite groups significantly associated with PA. Glycerophospholipids (28%), amino acids (15%), and glycerolipids (8%) were the most common metabolite groups associated with an increase in PA. Among glycerophospholipids phosphatidylcholines (39%), lysophosphatidylcholines (22%), and choline plasmalogens (9%) were the most frequent metabolites associated with PA. 

**Figure 3:**
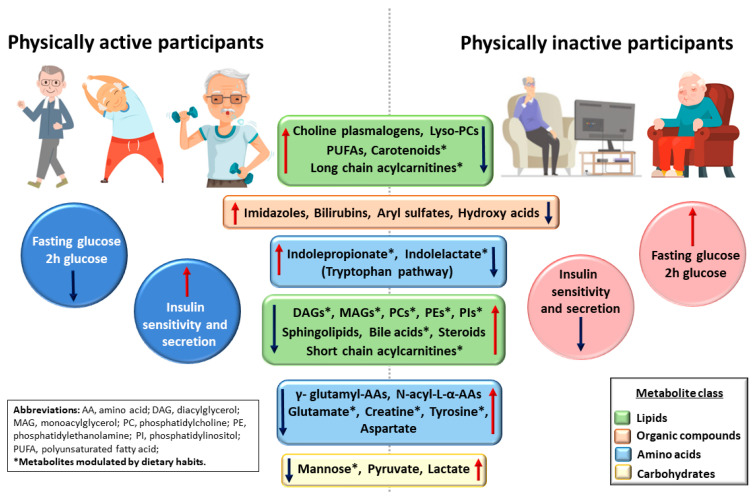
Physically active (PA3 or PA4) participants showed a decrease in fasting and 2 h glucose, and an increase in insulin sensitivity and insulin secretion compared to physically inactive participants. Physically active (PA3 or PA4) participants had increased levels of plasmalogen-cholines, lysoplasmalogencholines, polyunsaturated fatty acids, carotenoids, long chain acylcarnitines, imidazoles, bilirubins, aryl sulfates, hydroxy acids, indolepropionate, and indolelactate, and lower levels of diacylglycerols, monoacylglycerols, phosphatidylcholines, phosphatidylethanolamines, phosphatidylinositols, sphingolipids, bile acids, steroids, short-chain acyl carnitines, γ-glutamyl-amino acids, *N*-acyl-l-α-amino acids, glutamate, creatine, tyrosine, aspartate, mannose, pyruvate, and lactate than physically inactive participants.

Figure 3 and Table S1 show the individual metabolites that are significantly associated with PA. We found 198 metabolites significantly associated with PA. Several of these metabolites were novel, including especially steroids, amino acids, imidazoles, carboxylic acids, and hydroxy acids (Table S1). Of these metabolites, steroids and amino acids were increased and imidazoles, carboxylic acids, and hydroxy acids decreased in the physically active participants.

We found that participants in the PA+ group had higher levels of choline plasmalogens, lysophosphatidylcholines, polyunsaturated fatty acids (PUFA), long chain acylcarnitines, carotenoids, imidazoles, bilirubins, aryl sulfates, hydroxy acids, indolepropionate, and indolelactate, than participants in the PA− group. Diacylglycerols, monoacylglycerols, phosphatidylcholines, phosphatidylethanolamines, phosphatidylinositols, sphingolipids, bile acids, steroids, short-chain acylcarnitines, gamma-glutamyl-amino acids, glutamate, creatine, tyrosine, mannose, pyruvate, and lactate were decreased in the PA+ group compared to the PA− group. 

## 4. Discussion

Our results based on the large randomly selected population-based METSIM cohort showed that increased leisure-time PA was associated with a lower incidence of type 2 diabetes and increased insulin sensitivity and insulin secretion. Importantly, the metabolite signature of PA was significantly different between the PA+ and PA− groups.

Increased leisure-time PA decreases the conversion to type 2 diabetes based on several observational [4] and lifestyle intervention studies [17,18,19,20]. In the Finnish Diabetes Prevention study, PA of at least 2.5 h/week reduced the risk of type 2 diabetes by 62% in the intervention group but only 46% in the control group [21]. The results of our population-based study were quite similar, because in the participants engaging in PA at comparable activity level the conversion to type 2 diabetes was reduced by 39%. In the participants engaging in PA ≤ 2 times a week, the preventive effect was 30%. 

We applied a questionnaire previously validated in the Mini-Finland Health Survey to measure PA [14]. The participants were divided into four categories based on the frequency of leisure-time PA. We found that fasting and 2-h glucose and insulin concentrations decreased significantly and linearly from the lowest to the highest PA category. Similar beneficial linear trends were also observed in BMI, alcohol consumption and smoking, suggesting that PA reflects other actions promoting a healthy lifestyle [14]. 

In our study, insulin sensitivity (Matsuda ISI) and insulin secretion (Disposition index) increased linearly across the PA categories at baseline. We also showed that an increase in PA during the follow-up decreased fasting and 2-h glucose levels, and increased insulin sensitivity and insulin secretion compared to participants who did not increase their PA. Several studies have reported positive effects of aerobic exercise, resistance training, and their combination on insulin sensitivity [22,23,24,25], but the results with respect to insulin secretion have not been consistent across the studies. In the Diabetes Prevention Program, both decreased insulin sensitivity and insulin secretion were independently associated with the conversion to type 2 diabetes [26], but in other studies, the role of insulin secretion has remained unclear [27]. The validity of the measurement of insulin sensitivity and insulin secretion is crucial for obtaining reliable results. We measured insulin sensitivity and insulin secretion using validated methods [12]. Conflicting results on the role of PA in insulin secretion in previous studies may be due to the use of poor methods to measure insulin secretion. 

We measured the association of 1260 metabolites with PA in 7271 men. Our study is the largest in size and includes more metabolites than any previous study aiming to characterize the metabolite signature of PA. We found 198 metabolites significantly associated with PA. Several of these metabolites were novel including especially steroids, amino acids, imidazoles, carboxylic acids, and hydroxy acids. We found that the participants belonging to the two highest categories of PA had higher levels of choline plasmalogens, lysophosphatidylcholines, PUFA*s*, carotenoids, bilirubins, indolelactate, and indolepropionate than participants belonging to the two lowest categories of PA (Figure 3, Table S1). Previous smaller studies have also reported that choline plasmalogens and lysophosphatidylcholines are associated with a decreased risk of type 2 diabetes, decreased glucose levels, and increased insulin sensitivity [28,29,30,31]. Similarly, high concentrations of omega-3 and omega-6 PUFAs [32,33], carotenoids [34], hyperbilirubinemia [35], and indolepropionate have been associated with reduced risk of type 2 diabetes, and indolepropionate has also been associated with increased insulin secretion [36]. 

We found that the concentrations of several metabolites, including diacylglycerols, monoacylglycerols, phosphatidylcholines, phosphatidylethanolamines, phosphatidylinositols, sphingolipids, bile acids, and short-chain acylcarnitines, were decreased in the participants in the two highest PA groups. Our findings were largely novel, but elevated concentrations of diacylglycerols and phosphatidylethanolamines have been previously reported to increase the risk of type 2 diabetes [30]. Gamma-glutamyl-amino acids, N-acyl-L-alpha-amino acids, glutamate, creatine, tyrosine, aspartate, mannose, and glycolysis metabolites (lactate and pyruvate) were also decreased in the two highest PA groups compared to the two lowest PA groups. 

High concentrations of ceramides, which belong to the sphingolipid class, induce lipotoxicity and insulin resistance and are associated with increased risk of type 2 diabetes [37]. Elevated concentrations of short-chain acylcarnitines have been linked to insulin resistance [38]. A bile acid, glycodeoxycholic acid, has been associated with increased risk of type 2 diabetes [39]. Our previous study based on the METSIM cohort showed that tyrosine, aspartate, and glutamate were significantly associated with decreases in insulin sensitivity and insulin secretion and consequently with increased risk of type 2 diabetes [15]. Mannose is deposited as glycogen in the liver, and we have shown that a high mannose concentration increases the risk of type 2 diabetes [40]. 

Several metabolites associated with high PA were also associated with diet in previous publications (Figure 2). Bouchard-Mercier et al. reported that a Western diet was associated with short-chain acylcarnitines, whereas a prudent diet (high in vegetables, fruits, and whole-grain products) was associated with medium- to long-chain acylcarnitines [41]. A healthy diet, including whole grains, fatty fish, and bilberries (*Vaccinium myrtillus*) increases PUFA levels [42]. Carotenoid concentrations correlate with self-reported dietary intake of carotenoids and also with a decreased risk of type 2 diabetes [43]. Bile acid concentrations are also influenced by diet. A study by Sonne et al. showed that a high fat meal increased concentrations of bile acids [44]. Meikle et al. reported that men who consumed dairy fat had significantly increased lipids, including phosphatidylcholines, phosphatidylethanolamines, and phosphatidylinositols [45]. 

In summary, we found that the participants in the PA+ group had a healthier metabolite profile and lower risk of type 2 diabetes than participants in the PA− group. These results are consistent with previous studies showing that a healthy diet is associated with a metabolite signature similar to high PA. Beyond PA, dietary modification is another very important lifestyle change for subjects with the metabolic syndrome that has been shown to significantly decrease their risk of cardiovascular diseases [46]. Importantly, the metabolites differing between the two PA groups are dependent not only on diet but also on gut microbiota. About 70% of choline, which is the building block of phosphatidylcholines, lyso-phosphatidylcholines, and choline plasmalogens, is obtained from the diet [47] and is modulated by microbiota in the gut [48]. Similarly, diet and gut microbiota modulate PUFAs, acylcarnitines, carotenoids, tryptophan (precursor of indolelactate and indolepropionate) and bile acids [49]. Both PA and diet affect gut microbiota composition, and result in different metabolic profiles [50]. Our findings emphasize the importance of novel biomarkers for the management and treatment of the metabolic syndrome [51].

The strengths of our study are the large METSIM cohort, large number of cases with incident type 2 diabetes, and a long follow-up period. We measured insulin sensitivity and insulin secretion with the best surrogate markers available. Additionally, we measured 1290 metabolites from 7271 participants, indicating that our study had an excellent power to obtain reliable results on the association of PA with metabolites. As far as we know, our study is the largest population-based study investigating the association of PA with metabolite signature. 

We acknowledge certain limitations of our study. Measurements of PA in our study was self-reported, and the questionnaire included only questions on the frequency and duration of PA. No information about the intensity (light, moderate, vigorous) or type of PA was collected. Our questionnaire also lacked separation of those who exercised at the minimum level recommended to prevent type 2 diabetes, namely at least 150 min a week of PA [5]. However, although our questionnaire has limitations, self-reported PA correlates well with objectively measured PA [52], and by using this PA questionnaire we managed to detect significant differences in glucose levels, insulin sensitivity and insulin secretion between the PA categories. Further limitations of our study are that all participants were middle-aged and elderly Finnish men. Therefore, we do not know if our results are valid among women and in other populations. We were not able to replicate our results in other populations because large population-based studies having metabolomics data are not available. Replication of our novel results suggesting that a metabolite signature of PA includes also steroids, amino acids, imidazoles, carboxylic acids, and hydroxy acids is especially important.

## 5. Conclusions

In conclusion, our population-based study shows that a metabolite signature of increased physical activity includes multiple metabolic pathways and is associated with better adherence to a healthy lifestyle. 

## Figures and Tables

**Table 1:** Baseline clinical characteristics of the participants of the METSIM study without diabetes (*n* = 8749) in the categories of physical activity (PA).

	Leisure-Time Physical Activity Categories					
	PA1 (*n* = 537)	PA2 (*n* = 2517)	PA3 (*n* = 1498)	PA4 (*n* = 4197)	All (*n* = 8749)	
Variables	Mean ± SD	Mean ± SD	Mean ± SD	Mean ± SD	Mean ± SD	*p*-Value
Age, years	57.1 ± 6.9	57.4 ± 7.0	56.0 ± 6.7	57.5 ± 7.2	57.2 ± 7.1	<0.001
BMI, kg/m^2^	29.1 ± 5.6	27.4 ± 4.0	27.1 ± 3.8	26.1 ± 3.2	26.8 ± 3.8	<0.001
Waist, cm	104.5 ± 14.0	100.0 ± 10.7	98.1 ± 10.3	94.8 ± 9.2	97.4 ± 10.6	<0.001
Total alcohol consumption, g/wk	132.0 ± 187.0	103.0 ± 139.0	98.0 ± 114.0	89.0 ± 116.0	97.0 ± 129.0	<0.001
Current smokers, %	36.5	24.1	16.8	12.9	18.2	<0.001

Data are mean ± SD, current smoking %. *p* values comparing PA over the four PA groups were calculated by a one-way ANOVA, and smoking by a χ^2^ test. *p* < 0.010 is statistically significant given five variables included in the analyses. PA1 = physical activity a little or none, PA2 = physical activity in context of other hobbies or physical activity occasionally, PA3 = physical activity regularly ≤2 times a week at least 30 min at a time, PA4 = physical activity regularly ≥3 times a week at least 30 min at a time.

**Table 2:** Baseline characteristics of glucose and insulin concentrations, insulin sensitivity, and insulin secretion in the METSIM study in the categories of physical activity (PA) (*n* = 8749).

Physical Activity	PA1	PA2	PA3	PA4	Unadjusted *p*	Adjusted *p* *
Fasting glucose	5.79 ± 0.53	5.75 ± 0.48	5.73 ± 0.48	5.68 ± 0.47	<0.001	0.030
2-h glucose	6.54 ± 1.90	6.20 ± 1.71	6.08 ± 1.69	5.88 ± 1.62	<0.001	<0.001
Fasting insulin	11.50 ± 8.99	9.08 ± 6.50	8.49 ± 5.94	7.22 ± 4.58	<0.001	<0.001
2-h insulin	75.29 ± 68.44	57.99 ± 56.28	54.57 ± 54.53	44.42 ± 44.97	<0.001	<0.001
Matsuda ISI	5.32 ± 3.97	6.26 ± 3.92	6.70 ± 4.08	7.58 ± 4.21	<0.001	<0.001
DI	151.6 ± 74.2	157.8 ± 68.6	161.5 ± 69.1	169.2 ± 74.0	<0.001	<0.001

Abbreviations: ISI, insulin sensitivity index; DI, disposition index; *p-*values were calculated using linear regression. *p* < 0.008 is statistically significant given six variables included in analyses. * Adjusted for age, smoking, alcohol consumption, and BMI at baseline. Physical activity categories as in Table 1.

**Table 3:** The association of baseline PA with incident type 2 diabetes. The mean length of the follow-up time was 7.8 years.

			Unadjusted			Adjusted *		
Physical Activity Category	Total (*n* = 8749)	Incident Diabetes, (*n* = 1151)	HR	95% CI	*p*	HR	95% CI	p *
PA1	537	102 (19.0%)	1.00			1.00		
PA2	2517	401 (15.9%)	0.87	0.70, 1.08	0.213	1.15	0.92, 1.44	0.22
PA3	1498	204 (13.6%)	0.70	0.55, 0.89	0.004	1.03	0.81, 1.31	0.82
PA4	4195	444 (10.6%)	0.61	0.49, 0.75	<0.001	0.99	0.79, 1.24	0.93

* Adjusted for age, BMI, smoking, and alcohol consumption. Physical activity categories as in Table 1.

## Data Availability

The data presented in this study are available on request from the corresponding author. The data are not publicly available due to preserving the confidentiality of the participants.

## Supplementary Materials

The following supporting information can be downloaded at: https://www.mdpi.com/2218-1989/12/1/69/s1. Supplementary Table S1. Statistically significant (*p* < 3.9 × 10^−5^) differences in metabolite groups of the participants having low physical activity (Group 1, little or no physical activity or physical activity in the context of other hobbies) and high physical activity (Group 2, physical activity regularly ≤2 times a week at least 30 min at a time or physical activity regularly ≥3 times a week at least 30 min at a time).
