# Supplementary material for: Metabolite Signature of Physical Activity and the Risk of Type 2 Diabetes in 7271 Men
Source: Metabolites. 2022 Jan 12;12(1):69. doi: 10.3390/metabo12010069 (PMC8779070; doi:10.3390/metabo12010069)
Supplement: Supplementary file 1 [file metabolites-12-00069-s001.zip › metabolites-1535077-supplementary.pdf]

Table S1. Statistically significant ($P<3.9 \times 10^{-05}$) differences in metabolite groups of the participants having low physical activity (Group 1, a little or no physical activity or physical activity in context of other hobbies) and high physical activity (Group 2, physical activity regularly ≤2 times a week at least 30 min at a time or physical activity regularly ≥3 times a week at least 30 min at a time).

METABOLITE*	SUBCLASS	GROUP	N	Mean	SD	P**
**Lipids and lipid-like molecules**						
**Carotenoids**						
Beta-Cryptoxanthin	Tetraterpenoids	1	2408	-0,088	0,400	2,01E-33
		2	4699	0,027	0,369	
Carotenediol(2)	Tetraterpenoids	1	2474	-0,040	0,198	1,02E-27
		2	4778	0,012	0,188	
Carotenediol(1)	Tetraterpenoids	1	2484	-0,036	0,190	1,22E-22
		2	4787	0,009	0,182	
Carotenediol(3)	Tetraterpenoids	1	1980	-0,031	0,253	8,54E-10
		2	3776	0,012	0,247	
**Glycerolipids**						
Oleoyl-Oleoyl-Glycerol(18:1/18:1)[2]*	Diacylglycerol	1	1686	0,049	0,250	1,50E-20
		2	3110	-0,021	0,248	
Oleoyl-Oleoyl-Glycerol(18:1/18:1)[1]*	Diacylglycerol	1	1542	0,039	0,259	3,21E-15
		2	2779	-0,025	0,256	
Palmitoyl-Oleoyl-Glycerol(16:0/18:1)[2]*	Diacylglycerol	1	1372	0,048	0,296	4,73E-15
		2	2479	-0,028	0,280	
Palmitoleoyl-Oleoyl-Glycerol(16:1/18:1)[2]*	Diacylglycerol	1	1459	0,049	0,348	1,53E-13
		2	2739	-0,033	0,338	
Oleoyl-Arachidonoyl-Glycerol(18:1/20:4)[1]*	Diacylglycerol	1	2391	0,028	0,229	2,26E-13
		2	4607	-0,014	0,227	
Diacylglycerol(14:0/18:1,16:0/16:1)[1]*	Diacylglycerol	1	1240	0,054	0,330	4,13E-13
		2	2311	-0,030	0,324	
Diacylglycerol(14:0/18:1,16:0/16:1)[2]*	Diacylglycerol	1	1252	0,057	0,339	4,22E-13

		2	2366	-0,027	0,326	
Myristoyl-Linoleoyl-Glycerol(14:0/18:2)[2]*	Diacylglycerol	1	1634	0,046	0,336	9,13E-12
		2	2939	-0,024	0,333	
Palmitoleoyl-Oleoyl-Glycerol(16:1/18:1)[1]*	Diacylglycerol	1	1221	0,042	0,370	1,35E-11
		2	2147	-0,046	0,359	
Palmitoyl-Linoleoyl Glycerol(16:0/18:2)[2]*	Diacylglycerol	1	2174	0,021	0,268	2,48E-11
		2	4148	-0,027	0,268	
Palmitoyl-Oleoyl-Glycerol(16:0/18:1)[1]*	Diacylglycerol	1	938	0,051	0,285	3,86E-11
		2	1751	-0,024	0,274	
Palmitoleoyl-Linoleoyl Glycerol(16:1/18:2)[1]*	Diacylglycerol	1	2071	0,025	0,267	1,69E-10
		2	3983	-0,020	0,259	
Diacylglycerol(16:1/18:2[2],16:0/18:3[1]	Diacylglycerol	1	1923	0,024	0,289	5,39E-09
		2	3448	-0,023	0,288	
Oleoyl-Arachidonoyl - Glycerol(18:1/20:4)[2]*	Diacylglycerol	1	2277	0,018	0,252	9,09E-09
		2	4310	-0,019	0,245	
1-Palmitoleoylglycerol(16:1)*	Monoacylglycerol	1	2401	0,038	0,260	2,20E-14
		2	4605	-0,012	0,255	
1-Oleoylglycerol(18:1)	Monoacylglycerol	1	2373	0,030	0,229	1,02E-13
		2	4580	-0,011	0,215	
**Glycerophospholipids**						
1-Stearoyl-2-Docosapentaenoyl-GPC(18:0/22:5n6)*	Phosphatidylcholine	1	2465	0,033	0,194	7,82E-33
		2	4745	-0,025	0,195	
1-Stearoyl-2-Adrenoyl-GPC(18:0/22:4)*	Phosphatidylcholine	1	2477	0,033	0,185	1,52E-32
		2	4771	-0,022	0,184	
1-Stearoyl-2-Dihomo-Linolenoyl-GPC(18:0/20:3n3or6)*	Phosphatidylcholine	1	2481	0,018	0,130	4,20E-28
		2	4775	-0,018	0,132	
1-Stearoyl-2-Meadoyl-GPC(18:0/20:3n9)*	Phosphatidylcholine	1	2373	0,042	0,250	1,33E-25
		2	4579	-0,025	0,251	

1-Palmitoyl-2-Meadoyl-GPC(16:0/20:3n9)*	Phosphatidylcholine	1	1808	0,039	0,221	1,42E-20
		2	3436	-0,021	0,220	
1-Stearoyl-2-Oleoyl-GPC(18:0/18:1)	Phosphatidylcholine	1	2478	0,017	0,117	8,27E-18
		2	4770	-0,007	0,113	
1-Palmitoyl-2-Palmitoleoyl-GPC(16:0/16:1)*	Phosphatidylcholine	1	2484	0,034	0,206	2,04E-17
		2	4784	-0,008	0,199	
1-Palmitoyl-2-Adrenoyl-GPC(16:0/22:4)*	Phosphatidylcholine	1	2478	0,015	0,181	6,11E-17
		2	4766	-0,023	0,183	
1-Stearoyl-2-Arachidonoyl-GPC(18:0/20:4)	Phosphatidylcholine	1	2482	0,010	0,100	5,55E-15
		2	4783	-0,010	0,104	
1-Margaroyl-2-Docosahexaenoyl-GPC(17:0/22:6)*	Phosphatidylcholine	1	2232	-0,041	0,217	1,40E-14
		2	4453	0,001	0,210	
1-Palmitoyl-2-Arachidonoyl-GPC(16:0/20:4n6)	Phosphatidylcholine	1	2482	0,007	0,091	4,47E-14
		2	4785	-0,010	0,092	
1-Oleoyl-2-Docosahexaenoyl-GPC(18:1/22:6)*	Phosphatidylcholine	1	2480	-0,018	0,119	1,62E-13
		2	4773	0,003	0,112	
1-Palmitoyl-2-Oleoyl-GPC(16:0/18:1)	Phosphatidylcholine	1	2481	0,009	0,084	4,60E-12
		2	4777	-0,005	0,082	
1-Linoleoyl-2-Linolenoyl-GPC(18:2/18:3)*	Phosphatidylcholine	1	2454	-0,027	0,212	1,98E-11
		2	4751	0,009	0,210	
1-Pentadecanoyl-2-Docosahexaenoyl-GPC(15:0/22:6)*	Phosphatidylcholine	1	2454	-0,032	0,195	5,54E-11
		2	4729	-0,001	0,185	
Phosphatidylcholine(15:0/18:1,17:0/16:1, 16:0/17:1)*	Phosphatidylcholine	1	2478	0,009	0,141	3,75E-10
		2	4770	-0,013	0,134	
1-Oleoyl-2-Dihomo-Linolenoyl-GPC(18:1/20:3)*	Phosphatidylcholine	1	2404	0,005	0,138	7,25E-10
		2	4663	-0,016	0,135	
1,2-Dilinoleoyl-GPC(18:2/18:2)	Phosphatidylcholine	1	2464	-0,023	0,161	8,51E-10
		2	4735	0,001	0,153	
	Phosphatidylcholine	1	2484	-0,011	0,136	9,08E-10

Glycerophosphorylcholine(GPC)		2	4787	0,009	0,136	
1-Palmitoyl-2-Dihomo-Linolenoyl-GPC(16:0/20:3n3or6)*	Phosphatidylcholine	1	705	0,016	0,117	2,80E-09
		2	1508	-0,019	0,138	
1-Myristoyl-2-Arachidonoyl-GPC(14:0/20:4)*	Phosphatidylcholine	1	2476	0,009	0,198	1,84E-08
		2	4769	-0,018	0,198	
1-(1-Enyl-Palmitoyl)-2-Linoleoyl-GPC(P-16:0/18:2)*	Choline-Plasmalogen	1	2477	-0,023	0,132	1,87E-25
		2	4770	0,009	0,119	
1-(1-Enyl-Stearoyl)-2-Docosahexaenoyl-GPC(P-18:0/22:6)*	Choline-Plasmalogen	1	2473	-0,031	0,163	5,78E-25
		2	4768	0,010	0,157	
1-(1-Enyl-Palmitoyl)-2-Docosahexaenoyl-GPC(P-16:0/22:6)*	Choline-Plasmalogen	1	2480	-0,029	0,153	1,41E-17
		2	4774	0,003	0,146	
1-(1-Enyl-Stearoyl)-2-Linoleoyl-GPC(P-18:0/18:2)*	Choline-Plasmalogen	1	2459	-0,027	0,174	7,65E-16
		2	4755	0,006	0,158	
1-Palmityl-2-Oleoyl-GPC(O-16:0/18:1)*	Choline-Plasmalogen	1	2476	-0,013	0,097	5,30E-13
		2	4769	0,004	0,092	
1-(1-Enyl-Palmitoyl)-2-Oleoyl-GPC(P-16:0/18:1)*	Choline-Plasmalogen	1	2479	-0,011	0,118	6,74E-09
		2	4770	0,005	0,107	
1-Docosahexaenoyl-GPC(22:6)*	Lyso-phosphatidylcholine	1	2484	-0,030	0,183	8,44E-20
		2	4787	0,009	0,169	
1-Eicosenoyl-GPC(20:1)*	Lyso-phosphatidylcholine	1	2479	-0,014	0,133	3,59E-19
		2	4775	0,015	0,130	
1-Linoleoyl-GPC(18:2)	Lyso-phosphatidylcholine	1	2483	-0,020	0,125	1,42E-17
		2	4787	0,005	0,112	
1-Nonadecanoyl-GPC(19:0)	Lyso-phosphatidylcholine	1	2291	-0,029	0,175	3,82E-18
		2	4486	0,008	0,161	
1-Arachidoyl-GPC(20:0)	Lyso-phosphatidylcholine	1	2442	-0,022	0,146	4,29E-17
		2	4712	0,008	0,139	
2-Docosahexaenoyl-GPC(22:6)*		1	2278	-0,035	0,205	5,30E-15

	Lyso-phosphatidylcholine	2	4445	0,005	0,195	
2-Linoleoyl-GPC(18:2)*	Lyso-phosphatidylcholine	1	2466	-0,015	0,139	3,64E-13
		2	4761	0,009	0,135	
1-Lignoceroyl-GPC(24:0)	Lyso-phosphatidylcholine	1	2149	-0,022	0,154	5,54E-13
		2	4092	0,007	0,151	
1-Docosapentaenoyl-GPC(22:5n3)*	Lyso-phosphatidylcholine	1	2482	-0,022	0,161	4,37E-12
		2	4787	0,005	0,149	
1-Eicosapentaenoyl-GPC(20:5)*	Lyso-phosphatidylcholine	1	2483	-0,026	0,251	1,65E-11
		2	4787	0,015	0,242	
1-Erucoyl-GPC(22:1)*	Lyso-phosphatidylcholine	1	1898	0,006	0,242	8,98E-10
		2	3653	0,048	0,245	
1-Adrenoyl-GPC(22:4)*	Lyso-phosphatidylcholine	1	2472	0,010	0,171	1,06E-09
		2	4763	-0,016	0,178	
1-(1-Enyl-Palmitoyl)-GPC(P-16:0)*	Lysophosphatidyl-choline (Pl)	1	2480	-0,017	0,135	6,31E-15
		2	4781	0,009	0,131	
1-(1-Enyl-Stearoyl)-GPC(P-18:0)*	Lysophosphatidyl-choline (Pl)	1	2238	-0,021	0,182	1,71E-11
		2	4344	0,010	0,174	
1-(1-Enyl-Oleoyl)-GPC(P-18:1)*	Lysophosphatidyl-choline (Pl)	1	2437	-0,017	0,170	3,64E-11
		2	4710	0,010	0,164	
1-Stearoyl-2-Oleoyl-GPE(18:0/18:1)	Phosphatidyl-ethanolamine	1	2446	0,041	0,213	6,70E-26
		2	4710	-0,014	0,205	
1-Palmitoyl-2-Oleoyl-GPE(16:0/18:1)	Phosphatidyl-ethanolamine	1	2449	0,040	0,217	1,60E-22
		2	4724	-0,012	0,203	
1-Stearoyl-2-Arachidonoyl-GPE(18:0/20:4)	Phosphatidyl-ethanolamine	1	2474	0,022	0,164	5,78E-24
		2	4764	-0,016	0,153	
1-Palmitoyl-2-Arachidonoyl-GPE(16:0/20:4)*	Phosphatidyl-ethanolamine	1	2467	0,022	0,187	1,33E-14
		2	4756	-0,013	0,175	
1-Stearoyl-2-Linoleoyl-GPE(18:0/18:2)*		1	2464	0,028	0,211	6,10E-14

	Phosphatidyl-ethanolamine	2	4749	-0,010	0,199	
1-Palmitoyl-2-Palmitoleoyl-GPE(16:0/16:1)*	Phosphatidyl-ethanolamine	1	1640	0,060	0,307	2,63E-13
		2	2730	-0,007	0,288	
1-(1-Enyl-Oleoyl)-2-Docosahexaenoyl-GPE(P-18:1/22:6)*	Ethanolamine-Plasmalogen	1	2452	-0,027	0,182	3,10E-12
		2	4746	0,004	0,173	
1-(1-Enyl-Stearoyl)-2-Docosahexaenoyl-GPE(P-18:0/22:6)*	Ethanolamine-Plasmalogen	1	2471	-0,021	0,149	7,29E-11
		2	4761	0,003	0,144	
1-Palmitoleoyl-GPE(16:1)*	Lysophosphatidyl-ethanolamine	1	2066	0,042	0,279	1,71E-10
		2	3756	-0,007	0,276	
1-Palmitoyl-2-Oleoyl-GPI(16:0/18:1)*	Phosphoinositol	1	2432	0,023	0,187	2,08E-11
		2	4683	-0,007	0,175	
1-Palmitoleoyl-GPI(16:1)*	Phosphoinositol	1	1841	0,041	0,329	5,62E-09
		2	3278	-0,014	0,322	
1-Palmitoyl-2-Arachidonoyl-GPI(16:0/20:4)*	Phosphoinositol	1	2353	0,016	0,161	1,09E-08
		2	4482	-0,008	0,162	
1-Stearoyl-GPG(18:0)	Lyso-phosphoglycerol	1	1747	0,020	0,172	7,36E-10
		2	3205	-0,011	0,171	
1-Oleoyl-GPG(18:1)*	Lyso-phosphoglycerol	1	1658	0,017	0,171	3,05E-08
		2	3309	-0,012	0,169	
**Fatty acids and fatty acids derivatives**						
Hydroxy-CMPF*	Furanoid fatty acid derivative	1	2484	-0,108	0,375	3,46E-30
		2	4787	-0,010	0,332	
3-Carboxy-4-Methyl-5-Propyl-2-Furanpropanoate(CMPF)	Furanoid fatty acid derivative	1	2484	-0,105	0,431	3,67E-12
		2	4787	-0,037	0,382	
Docosahexaenoate(DHA;22:6n3)	PUFA- Very long chain fatty acid	1	2484	-0,036	0,221	7,01E-19
		2	4787	0,011	0,210	
Docosapentaenoate (N3dpa;22:5n3)	PUFA- Very long chain fatty acid	1	2484	-0,020	0,199	5,44E-11
		2	4787	0,012	0,197	

Erucate(22:1n9)	Very long chain fatty acid	1	2484	-0,007	0,219	6,66E-09
		2	4787	0,025	0,224	
2-Hydroxyarachidate*	Long chain fatty acids	1	2364	0,016	0,125	1,43E-15
		2	4545	-0,009	0,118	
Eicosapentaenoate(EPA;20:5n3)	PUFA - Long chain fatty acid	1	2484	-0,027	0,236	3,28E-14
		2	4787	0,017	0,230	
Linolenate[Alphaorgamma; (18:3n3or6)]	PUFA - Long chain fatty acid	1	2484	-0,026	0,193	8,14E-10
		2	4787	0,003	0,192	
Stearidonate(18:4n3)	PUFA - Long chain fatty acid	1	2483	-0,017	0,221	1,14E-08
		2	4787	0,014	0,218	
(2or3)-Decenoate(10:1n7orn8)	Medium chain fatty acids	1	2456	-0,030	0,181	1,23E-17
		2	4740	0,009	0,184	
3-Hydroxydecanoate	3-hydroxyacyl CoAs	1	2484	-0,022	0,170	6,24E-16
		2	4787	0,013	0,173	
Docosahexaenoylcholine	Acyl choline	1	2454	-0,039	0,277	3,79E-11
		2	4742	0,005	0,261	
Heneicosapentaenoate(21:5n3)	PUFA – Long chain fatty acid	1	2196	-0,054	0,382	3,94E-11
		2	4461	0,012	0,376	
**Sphingolipids**						
Sphingomyelin(D18:0/18:0,D19:0/17:0)*	Sphingomyelin	1	2475	0,039	0,209	1,32E-28
		2	4762	-0,018	0,207	
N-Stearoyl-Sphingosine(D18:1/18:0)*	Sphingomyelin	1	1884	0,024	0,150	2,50E-26
		2	3466	-0,023	0,152	
Sphingomyelin(D18:0/20:0,D16:0/22:0)*	Sphingomyelin	1	2450	0,022	0,187	4,93E-15
		2	4704	-0,015	0,192	
Hexadecasphingosine(D16:1)*	Sphingomyelin	1	1820	0,014	0,166	6,20E-10
		2	3347	-0,015	0,161	
N-Palmitoyl-Sphinganine(D18:0/16:0)	Ceramide	1	2062	0,023	0,174	9,42E-16
		2	3828	-0,015	0,170	

N-Palmitoyl-Sphingosine(D18:1/16:0)	Ceramide	1	2484	0,011	0,109	1,13E-13
		2	4787	-0,008	0,104	
N-Stearoyl-Sphinganine(D18:0/18:0)*	Ceramide	1	1196	0,036	0,280	6,20E-13
		2	1952	-0,038	0,281	
N-Stearoyl-Sphingadienine(D18:2/18:0)*	Ceramide	1	1947	0,011	0,185	1,10E-12
		2	3570	-0,027	0,188	
Ceramide(D18:1/20:0,D16:1/22:0,D20:1/ 18:0)*	Ceramide	1	1645	0,014	0,149	1,03E-10
		2	3063	-0,015	0,146	
Sphingosine1-Phosphate	Phosphosphingolipid	1	2483	0,008	0,116	7,75E-09
		2	4787	-0,008	0,109	
Sphinganine-1-Phosphate	Phosphosphingolipid	1	2313	0,008	0,153	1,43E-08
		2	4364	-0,014	0,147	
**Acyl Carnitines**						
Butyrylcarnitine(C4)	Acyl carnitine (short chain)	1	2479	0,052	0,214	8,28E-18
		2	4768	0,007	0,213	
Propionylcarnitine(C3)	Acyl carnitine (short chain)	1	2484	0,019	0,136	2,61E-17
		2	4787	-0,011	0,141	
Linolenoylcarnitine(C18:3)*	Acyl carnitine (long chain)	1	2333	-0,022	0,166	1,07E-12
		2	4536	0,008	0,163	
Hexanoylcarnitine(C6)	Acyl carnitine (short chain)	1	2484	0,021	0,174	2,11E-12
		2	4787	-0,009	0,167	
Linoleoylcarnitine(C18:2)*	Acyl carnitine (long chain)	1	2484	-0,012	0,133	1,46E-11
		2	4786	0,010	0,131	
Succinylcarnitine(C4-DC)	Acyl carnitine (short chain)	1	2468	0,011	0,139	2,06E-11
		2	4756	-0,012	0,138	
Isovalerylcarnitine(C5)	Acyl carnitine (short chain)	1	2484	0,010	0,147	1,32E-10
		2	4786	-0,013	0,143	
3-Hydroxydecanoylcarnitine	Acyl carnitine (medium chain)	1	2331	-0,028	0,202	5,73E-09
		2	4444	0,003	0,208	

Adipoylcarnitine(C6-DC)	Acyl carnitine (medium chain)	1	2464	0,029	0,212	1,27E-08
		2	4751	0,001	0,198	
2-Methylbutyrylcarnitine(C5)	Acyl carnitine (short chain)	1	2118	0,011	0,150	1,52E-08
		2	4042	-0,011	0,146	
**Bile Acids**						
Glycoursodeoxycholic acid sulfate(1)	Secondary bile acid	1	1551	0,076	0,436	3,62E-12
		2	2567	-0,017	0,402	
Glycoursodeoxycholate	Secondary bile acid	1	2427	0,054	0,474	5,19E-10
		2	4660	-0,018	0,450	
**Steroids**						
Cortoloneglucuronide(1)	Steroidal glycoside	1	2345	0,015	0,175	1,88E-21
		2	4458	-0,028	0,177	
Tetrahydrocortisol glucuronide	Steroidal taurinated bile acid	1	2459	0,007	0,176	1,16E-09
		2	4729	-0,019	0,174	
Pregnenetriol disulfate*	Pregnane steroid	1	2483	0,012	0,257	1,34E-09
		2	4787	-0,026	0,248	
Androstenediol(3beta,17beta)Disulfate(1)	Sulfated steroid	1	2484	0,036	0,349	1,12E-08
		2	4787	-0,010	0,319	
**Amino acids**						
Glutamate	-	1	2484	0,017	0,159	1,36E-18
		2	4787	-0,019	0,162	
Creatine	-	1	2484	0,022	0,190	4,92E-18
		2	4787	-0,019	0,189	
Tyrosine	-	1	2484	0,008	0,082	1,03E-08
		2	4785	-0,003	0,078	
Aspartate	-	1	2484	0,015	0,130	3,47E-15
		2	4786	-0,011	0,130	
N-Acetyltryptophan		1	2447	0,020	0,157	1,59E-17

	N-acyl-L-alpha-amino acids	2	4701	-0,013	0,152	
N-Acetylphenylalanine	N-acyl-L-alpha amino acid	1	2428	0,026	0,173	6,03E-12
		2	4665	-0,003	0,161	
N-Acetyltyrosine	N-acyl-L-alpha amino acid	1	2441	0,022	0,172	1,12E-11
		2	4675	-0,006	0,163	
Cinnamoylglycine	N-acyl-alpha amino acids	1	2285	-0,139	0,505	2,62E-10
		2	4495	-0,060	0,483	
Indolepropionate	Indole derivative (Tryptophan)	1	2458	-0,058	0,362	3,08E-17
		2	4742	0,018	0,360	
Indolelactate	Indole derivative (Tryptophan)	1	2484	-0,012	0,121	1,03E-08
		2	4787	0,004	0,115	
Gamma-Glutamylisoleucine*	Gamma-glutamyl amino acid	1	2481	0,026	0,173	2,78E-15
		2	4781	-0,007	0,168	
Gamma-Glutamylglutamate	Gamma-glutamyl-amino acid	1	2430	0,033	0,213	1,06E-12
		2	4668	-0,003	0,202	
Gamma-Glutamylvaline	Gamma-glutamyl amino acid	1	2480	0,023	0,170	3,14E-09
		2	4777	-0,001	0,159	
Gamma-Glutamylphenylalanine	Gamma-glutamyl amino acid	1	2483	0,011	0,094	6,47E-09
		2	4786	-0,003	0,094	
Formiminoglutamate	Glutamic acid and derivatives	1	1775	0,033	0,191	1,96E-14
		2	3262	-0,010	0,191	
Phenyllactate(PLA)	Phenylpropanoic acid (Phenylalanine)	1	2484	-0,012	0,125	1,91E-13
		2	4787	0,011	0,126	
Cysteines-Sulfate	L-alpha amino acid	1	2349	0,008	0,204	3,49E-13
		2	4545	-0,029	0,200	
5-Hydroxylysine	L-alpha amino acid	1	2424	0,018	0,130	6,78E-13
		2	4627	-0,005	0,125	
4-Hydroxyglutamate	L-alpha-amino acid	1	1915	0,013	0,213	9,43E-12

		2	3420	-0,028	0,210	
1-Carboxyethylphenylalanine	L-alpha amino acid	1	2468	0,019	0,166	1,60E-11
		2	4752	-0,007	0,154	
Stachydrine	L-alpha amino acid (Proline)	1	2484	-0,171	0,608	2,03E-11
		2	4787	-0,072	0,589	
Methioninesulfoxide	L-alpha-amino acid	1	2484	-0,004	0,106	9,93E-10
		2	4787	0,013	0,109	
N-Delta-Acetylornithine	L-alpha-amino acid	1	2481	-0,034	0,235	5,36E-09
		2	4786	0,000	0,231	
Cysteinesulfinicacid	L-alpha-amino acid	1	2160	0,012	0,141	7,77E-09
		2	4045	-0,010	0,142	
2-Oxoarginine*	Keto acid	1	2280	0,031	0,192	6,73E-11
		2	4352	-0,001	0,180	
Alpha-Ketoglutaramate*	Keto acid	1	2480	-0,007	0,101	8,20E-10
		2	4780	0,007	0,093	
Alpha-Ketoglutarate	Keto acid	1	2483	0,019	0,137	9,34E-10
		2	4785	0,000	0,118	
1-Carboxyethylvaline	Carboxylic acid (Valine)	1	2383	0,015	0,187	1,32E-10
		2	4585	-0,015	0,178	
1-Carboxyethylleucine	Carboxylic acid (Leucine)	1	2065	0,010	0,180	5,50E-10
		2	3876	-0,019	0,173	
Guanidinosuccinate	Carboxylic acid (Aspartate)	1	1447	-0,036	0,243	9,58E-10
		2	3169	0,010	0,227	
Carnitine	Carnitine	1	2482	0,005	0,064	1,56E-15
		2	4781	-0,007	0,064	
**Organic compounds**						
**Imidazoles**						
Imidazolelactate	Imidazole	1	2480	-0,021	0,139	5,90E-28

		2	4782	0,018	0,146	
1-Methyl-5-Imidazoleacetate	Imidazole	1	2462	-0,066	0,404	9,25E-16
		2	4748	0,013	0,393	
1-Ribosyl-Imidazoleacetate*	Imidazole	1	2478	-0,010	0,176	2,66E-09
		2	4780	0,015	0,171	
1-Methyl-5-Imidazolelactate	Imidazole	1	2318	-0,058	0,383	3,00E-09
		2	4548	-0,001	0,375	
**Aryl sulfates**						
O-Cresolsulfate	Aryl sulfate	1	2137	0,113	0,486	3,23E-17
		2	3859	0,006	0,459	
2-Aminophenolsulfate	Aryl sulfate	1	2476	-0,087	0,365	4,19E-11
		2	4780	-0,029	0,355	
Catecholsulfate	Aryl sulfate	1	2484	-0,047	0,298	1,01E-10
		2	4787	0,000	0,290	
4-Allylphenol sulfate	Aryl sulfate	1	2480	-0,024	0,418	4,69E-09
		2	4779	0,035	0,410	
Methyl-4-Hydroxybenzoatesulfate	Aryl sulfate	1	2328	-0,044	0,715	2,34E-08
		2	4607	0,058	0,713	
**Bilirubins**						
Bilirubin(Z,Z)	Bilirubin	1	2484	-0,015	0,181	2,77E-13
		2	4786	0,016	0,172	
Bilirubin(E,Zorz,E)*	Bilirubin	1	2107	-0,018	0,185	5,14E-11
		2	3930	0,014	0,177	
Biliverdin	Bilirubin	1	2483	-0,017	0,218	9,67E-10
		2	4787	0,016	0,211	
Bilirubin degradation product C17H18N2O4(2)**	-	1	2484	-0,023	0,217	1,27E-08
		2	4787	0,007	0,212	
	-	1	2484	-0,026	0,221	1,44E-08

Bilirubin degradation product C17H18N2O4(1)**		2	4787	0,004	0,214	
Bilirubin degradation product C16H18N2O5(2)**	-	1	2482	-0,025	0,242	2,83E-08
		2	4784	0,009	0,240	
**Carboxylic acids**						
Tartronate(Hydroxymalonate)	Dicarboxylic acid	1	2477	-0,051	0,205	6,19E-22
		2	4784	-0,006	0,177	
Oxalate(Ethanedioate)	Dicarboxylic acid	1	2484	-0,045	0,165	3,29E-21
		2	4787	-0,010	0,143	
Octadecadienedioate(C18:2-DC)*	Dicarboxylic acid	1	2478	-0,021	0,227	4,81E-13
		2	4782	0,018	0,220	
**Hydroxy acids**						
2R,3R-Dihydroxybutyrate	Hydroxy acid	1	2483	0,015	0,140	1,34E-11
		2	4785	-0,007	0,129	
3-Hydroxylaurate	Hydroxy acid	1	2484	-0,026	0,198	2,88E-11
		2	4785	0,007	0,201	
3-Hydroxyoctanoate	Hydroxy acid	1	2482	-0,018	0,171	1,04E-10
		2	4785	0,010	0,175	
3-Hydroxydodecanedioate*	Hydroxy acid	1	1750	-0,018	0,303	2,05E-09
		2	3541	0,035	0,308	
**Other organic compounds**						
2-Naphtholsulfate	Naphtalene	1	2351	0,124	0,443	7,45E-32
		2	4461	-0,002	0,404	
2,6-Dihydroxybenzoicacid	Benzoic acids and derivatives	1	2484	-0,049	0,305	3,44E-21
		2	4787	0,021	0,293	
2-Hydroxyfluorenesulfate	Fluorene	1	1667	0,090	0,385	1,52E-17
		2	2742	-0,010	0,372	
(2,4or2,5)-Dimethylphenolsulfate	Xylene	1	1855	0,076	0,467	2,57E-16
		2	3129	-0,036	0,470	

Cotinine	Pyrrolidinylpyridine	1	969	0,286	1,274	1,49E-11
		2	1462	-0,069	1,258	
4-Hydroxychlorothalonil	Benzenoid	1	2155	-0,031	0,281	2,04E-10
		2	4232	0,017	0,292	
Hydroxycotinine	Pyrrolidinylpyridine	1	665	-0,039	0,485	6,89E-10
		2	745	-0,217	0,581	
Pyridoxate	Pyridinecarboxylic acids	1	2484	0,010	0,220	6,13E-11
		2	4787	0,046	0,218	
Sphingosine	Amine	1	2424	0,021	0,190	2,63E-10
		2	4656	-0,008	0,179	
Sphinganine	Amine	1	2250	0,017	0,176	7,31E-09
		2	4289	-0,009	0,168	
5-Methylthioadenosine(MTA)	Nucleoside	1	2480	0,011	0,124	1,70E-09
		2	4784	-0,007	0,119	
Gamma-CEHC	Benzopyran	1	2473	-0,032	0,234	1,22E-08
		2	4773	0,001	0,232	
N-Acetylkynurenine(2)	Alkyl-phenylketones	1	2184	0,030	0,282	1,60E-08
		2	4105	-0,011	0,271	
**Carbohydrates and carbohydrates derivatives**						
Glycerate	Sugar acids and derivatives	1	2482	-0,041	0,140	1,22E-25
		2	4785	-0,008	0,122	
Threonate	Sugar acids and derivatives	1	2482	-0,026	0,206	5,82E-13
		2	4784	0,009	0,188	
Mannose	-	1	2483	0,009	0,098	2,90E-20
		2	4785	-0,013	0,094	
Methylglucopyranoside(Alpha+Beta)	O-glycosyl compounds	1	2303	-0,013	0,391	2,47E-11
		2	4577	0,053	0,381	
Pyruvate	-	1	2483	0,011	0,128	6,87E-11

		2	4784	-0,009	0,120	
N-Acetylglucosamine/N-Acetylgalactosamine	Acyl-aminosugars	1	2461	0,007	0,116	1,50E-10
		2	4710	-0,012	0,115	
Lactate	-	1	2483	0,013	0,114	4,69E-09
		2	4785	-0,002	0,106	
N-Acetylneuraminate	N-acylneuraminic acids	1	2482	0,014	0,123	3,04E-08
		2	4780	-0,002	0,118	
**Other metabolites**						
Branchedchain14:0dicarboxylicacid**	-	1	2448	-0,084	0,333	2,27E-24
		2	4736	-0,002	0,316	
Methylnaphthylsulfate(2)*	-	1	1566	0,152	0,503	2,77E-21
		2	2460	0,004	0,470	
Dodecadienoate(12:2)*	-	1	2484	-0,024	0,159	1,86E-17
		2	4787	0,009	0,156	
4-Methoxyphenolsulfate	-	1	2399	-0,082	0,357	4,02E-17
		2	4651	-0,007	0,350	
Mannonate*	-	1	2482	0,019	0,130	2,83E-11
		2	4783	-0,001	0,119	
Hydroxyasparagine**	-	1	2479	0,013	0,100	6,57E-11
		2	4776	-0,002	0,093	
Cis-3,4-Methyleneheptanoate	-	1	2469	-0,042	0,258	1,83E-10
		2	4774	-0,003	0,249	
2-Acetamidophenolsulfate	-	1	2043	-0,073	0,388	5,74E-10
		2	4079	-0,009	0,377	
Metaboloniclactonesulfate	-	1	2455	0,007	0,290	1,85E-09
		2	4746	-0,037	0,290	
Pentose acid*	-	1	2353	-0,014	0,240	6,73E-09
		2	4590	0,021	0,231	

Abbreviations: Pl, plasmalogen; PUFA, polyunsaturated fatty acid.
*200 most significant associations are shown; 1260 metabolites were included in the analysis. **P values were calculated using one-way ANOVA. Novel metabolites associated with physical exercise are underlined.
